# Optical and Electrochemical Properties of Self-Organized TiO_2_ Nanotube Arrays From Anodized Ti−6Al−4V Alloy

**DOI:** 10.3389/fchem.2019.00066

**Published:** 2019-02-08

**Authors:** Henia Fraoucene, Vinsensia Ade Sugiawati, Djedjiga Hatem, Mohammed Said Belkaid, Florence Vacandio, Marielle Eyraud, Marcel Pasquinelli, Thierry Djenizian

**Affiliations:** ^1^Laboratory of Advanced Technologies of Genie Electrics, Faculty of Electrical and Computer Engineering Mouloud Mammeri University, Tizi-Ouzou, Algeria; ^2^Electrochemistry of Materials Research Group, Aix-Marseille Université, CNRS, MADIREL, UMR 7246, Marseille, France; ^3^Optoelectronics and Photovoltaics (OPTO-PV) Team, Institute of Microelectronic Materials Nanosciences of Provence (IM2NP), St Jérôme Center, University of Provence, Marseille, France; ^4^Mines Saint-Etienne, Department of Flexible Electronics, Center of Microelectronics in Provence, Gardanne, France

**Keywords:** TiO_2_ nanotubes, Ti-6Al-4V alloy, anodization, Li-ion microbatteries, negative electrode

## Abstract

Due to their high specific surface area and advanced properties, TiO_2_ nanotubes (TiO_2_ NTs) have a great significance for production and storage of energy. In this paper, TiO_2_ NTs were synthesized from anodization of Ti-6Al-4V alloy at 60 V for 3 h in fluoride ethylene glycol electrolyte by varying the water content and further annealing treatment. The morphological, structural, optical and electrochemical performances of TiO_2_ NTs were investigated by scanning electron microscope (SEM), energy dispersive X-ray spectroscopy (EDS), X-ray diffraction (XRD), UV-Visible spectroscopy and electrochemical characterization techniques. By varying the water content in the solution, a honeycomb and porous structure was obtained at low water content and the presence of (α + β) phase in Ti-6Al-4V alloy caused not uniform etching. With an additional increase in water content, a nanotubular structure is formed in the (α + β) phases with different morphological parameters. The anatase TiO_2_ NTs synthesized with 20 wt% H_2_O shows an improvement in absorption band that extends into the visible region due the presence of vanadium oxide in the structure and the effective band gap energy (*Eg*) value of 2.25 eV. The TiO_2_ NTs electrode also shows a good cycling performance, delivering a reversible capacity of 82 mAh.g^−1^ (34 μAh.cm^−2^.μm^−1^) at 1C rate over 50 cycles.

## Introduction

Rechargeable batteries play an important role in powering the electronic devices and in storing energy due to their high energy and power density which are expected to be a solution for the future energy storage requirements (Li et al., 2017). Due to the lack of suitable on-board power sources, the advances in the miniaturization of microelectronics is growing, opening opportunities to explore the both cathode and anode materials as thin-films (Sugiawati et al., 2016) and nanostructured electrodes by utilizing various synthesis and deposition techniques (Djenizian et al., 2011; Pikul et al., 2013; Ellis et al., 2014; Xiong et al., 2014; Sugiawati et al., 2018). Titanium dioxide (TiO_2_) is a semiconductor material that has been studied extensively in the last few decades due to its chemical stability, non-toxicity and biocompatibility (Morozová et al., 2012; Pansila et al., 2012; Reszczynska et al., 2014). In Li-ion microbatteries, the electrochemical performances of anode materials are highly dependent on their morphologies, surface characteristics, and particle sizes. Many researchers proposed to reduce the size of TiO_2_ anode material to the nanometer scale in order to increase not only the number of reaction sites, but also gives new properties to the materials (Armstrong et al., 2005). Among these nanostructured materials, self-organized TiO_2_ nanotubes (TiO_2_ NTs) obtained by anodization of Ti foil can give a high porosity and larger specific area offering an enhancement in the cell capacity and cycle life (Ortiz et al., 2008, 2009; Fang et al., 2009; Panda et al., 2012; Kyeremateng et al., 2013b; Plylahan et al., 2014; Chang et al., 2015; Salian et al., 2017).

In addition, physical and electrochemical performance of TiO_2_ NTs can be enhanced by chemical modification of the surface (Plylahan et al., 2012; Kyeremateng et al., 2013a; Sopha et al., 2017) and by incorporation of foreign ions into TiO_2_ lattice such as Sn^4+^(Kyeremateng et al., 2013b), Fe^3+^(Das et al., 2011), Ni^2+^(Choi et al., 2009), Nb^5+^(Salian et al., 2018), and V^3+^(Lin et al., 2013). The electrical conductivity and electrochemical kintetics of TiO_2_ NTs electrodes can be improved by doping with Ti^3+^ ions due to a Li^+^ diffusion coefficient of 1.09 × 10^−12^ cm^2^ s^−1^ which is almost one order of magnitude higher than that of TiO_2_ NTs (1.39 × 10^−13^ cm^2^ s^−1^) (Duan et al., 2016). Yu et al. also demonstrated that 5 at.% Sn doped TiO_2_ NTs exhibits the best cycling stability with specific capacity of 386 mAh.g^−1^ and coulombic efficiency of 99.2% after 50 cycles at 0.1C (Yu et al., 2014).

Ternary titanium alloy (Ti-6Al-4V, with 6 wt% Aluminum and 4 wt% Vanadium) have also been utilized to synthesize the self-organized TiO_2_ NTs, notably for their use in a wide range of applications such as bone substitute applications, including orthopedic and dental implants due to their superior compatibility, mechanical resistance, excellent corrosion resistance, and good thermal stability (Long and Rack, 1998; Black and Hastings, 2013). Furthermore, research works focused to improve the osseointegration and stability of the TA6V implant in the human body (Jo et al., 2013). However, to the best of our knowledge, there have been no reports to date on the use of the anodized TA6V alloy as electrode for Li-ion microbatteries. The basic objective of this work is therefore to study the electrochemical performance and optical properties of the anodized TA6V alloy produced through electrochemical anodization in fluoride-containing ethylene glycol electrolyte.

## Experimental

### Synthesis of TiO_2_ Nanotubes

The Ti−6Al−4V (TA6V) alloy (0.1 mm thickness, 25% tolerance, Goodfellow) were cut into square shape (1.2 × 1.2 cm) with a selected work area of 0.6 cm^2^. Before anodization, the Ti−6Al−4V foils were degreased by sonication in acetone, 2-propanol and methanol for 10 min each, rinsed with ultrapure water and dried in a stream of compressed air. The anodization was performed in a two-electrode electrochemical cell with Ti−6Al−4V foil as the anode and platinum foil as the cathode. At room temperature, all anodization experiments were carried out under a constant voltage of 60 V using a generator (ISO-TECH IPS-603) for 3 h. Ethylene glycol (EG) solution containing 0.3 wt% ammonium fluoride (NH_4_F) was used as electrolyte, and the water content was varied at 2, 5, 10,15, and 20 wt%. After anodization, the samples were soaked in ultrapure water for 10 min and then dried in an oven at 50°C for 10 min. In order to transform the amorphous crystallographic structure obtained just after electrochemical anodization into crystalline structure, the samples were annealed at 500°C for 3 h with a heating and cooling rate of 5°C/min.

### Characterization of the Samples

Morphological characterization of the TiO_2_ NTs was investigated by scanning electron microcopy (SEM) using a PHILIPS XL30. The chemical composition was analyzed by energy dispersive X-ray spectroscopy (EDS). The crystalline phases were characterized by X-ray diffraction (XRD) analysis. The diffraction patterns were obtained by a X'Pert Philips MPD with a Panalytical X'Celerator detector using a graphite monochromized CuKα radiation (λ = 1.5418°A). The measurements were performed within the range of 2θ from 20° to 70°. The optical properties were investigated using a UV-Visible spectroscopy from 250 to 800 nm.

The electrochemical performance tests were performed using two-electrode Swagelok cells assembled in an argon-filled glove box in which the oxygen and moisture contents were <2 ppm. A 9 mm diameter Li foil was used as the counter electrode and two sheets of Whatman glass microfiber separator were soaked in the electrolyte of lithium hexafluorophospahte in ethylene carbonate and diethylene carbonate electrolyte (1M LiPF_6_ in EC/DEC of 1:1 w/w) purchased from Sigma-Aldrich prior to assembling the cell. The cycling experiments were performed using a VMP3 potentiostat-galvanostat (Biologic, France). For all experiments, no additives such as poly (vinyl difluoride) as binder and carbon black as conductive agent were utilized. Cyclic voltammetry (CV) measurements were performed in the range voltage of 1–3 V vs. Li/Li^+^ at a scan rate of 0.05, 0.1 and 0.5 mV.s^−1^, respectively. For galvanostatic discharge–charge tests, a constant current density of 3.23 μA.cm^−2^ (C/10), 6.47 μAcm^−2^ (C/5), 16.18 μA.cm^−2^ (C/2), and 32.35 μA.cm^−2^ (1C), respectively, was applied to the assembled cells with a cut-off potential of 1- 3 V vs. Li/Li^+^.

## Experimental Results

### Morphological and Chemical Characterization

#### Surface Morphology

**Figure 1:**
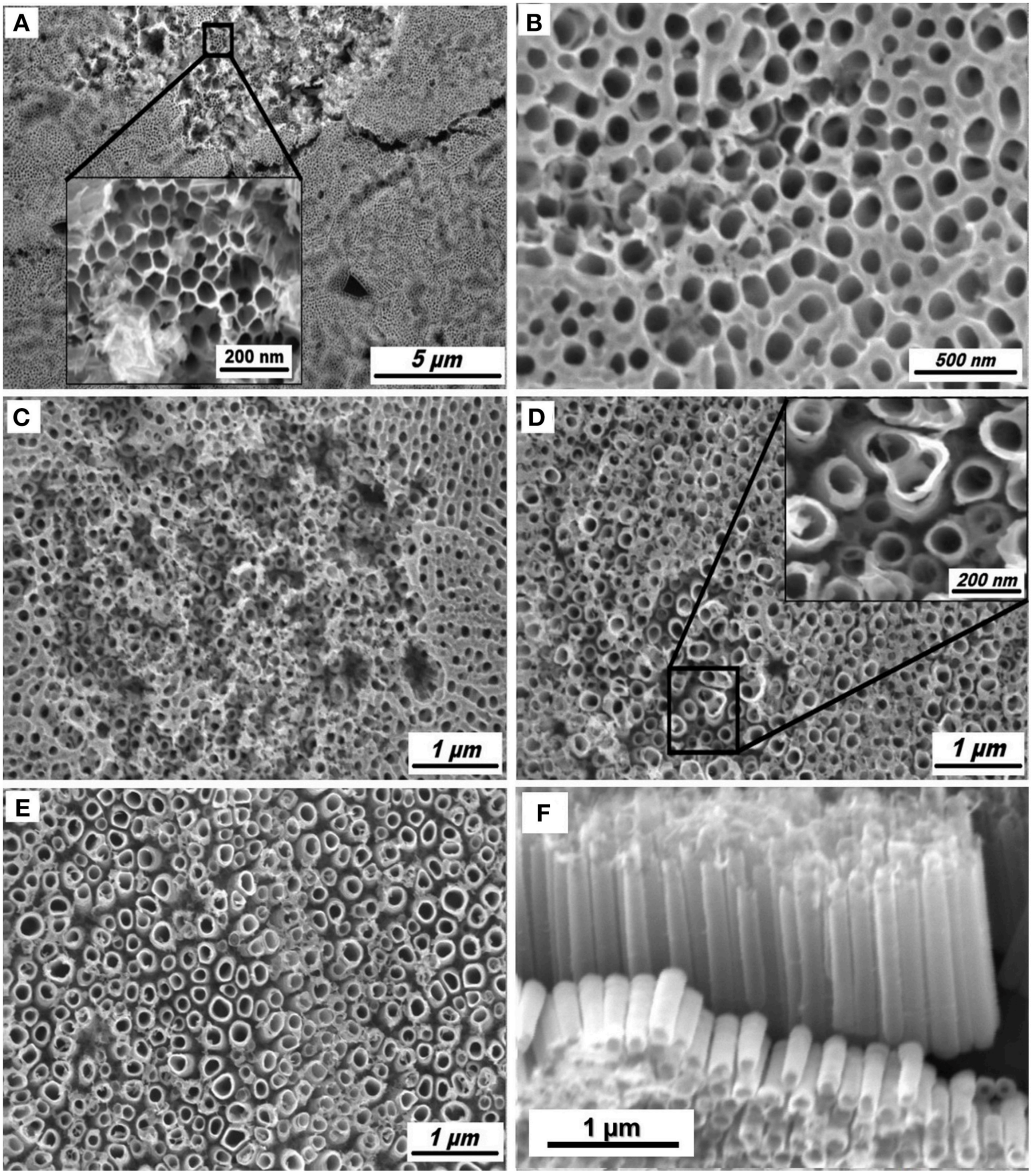
SEM images of TiO_2_ NTs obtained from anodization of TA6V alloy in fluoride ethylene glycol electrolyte with different water contents: 2 wt% **(A)**, 5 wt% **(B)**, 10 wt% **(C)**, 15 wt% **(D)**, and 20 wt% **(E)**. Tilted view of TiO_2_ NTs synthesized in 20 wt% H_2_O **(F)**.

Figure 1 shows the SEM images of the different morphologies of TA6V alloy anodized at 60 V for 3 h in Ethylene Glycol (EG) solution containing 0.3 wt% NH_4_F with different water contents. It should be noticed that the ternary titanium alloy studied in the present work consist of two metallurgical phases, the α phase being enriched in Al and the β phase in V (Macak et al., 2005). We noted a great influence of the water content on the formation of the self-organized TiO_2_ NTs, both randomly arranged porous structure and uniformly arranged nanotubes. The formation of the porous structure depends on the underlying phase (α or β) and the anodization parameters. As seen in Figure 1A, a honeycomb is obviously formed on the surface at water content of 2 wt%, however not on the entire surface. Figures 1B-D exhibits the positive influence of the improved water content, from 5 to 15 wt%. The β phase is preferentially etched as the amount of water in the solution increases, indicating the enhanced solubility of the vanadium oxides. A similar phenomenon has been previously reported for the TiO_2_ NTs grown from anodization of TA6V alloy in different electrolytes (sulphuric/hydrofluoric acid and ammonium sulfate with 0.2 wt% NH_4_F, respectively) at controlled voltage and anodization time (Matykina et al., 2011; Moravec et al., 2016). According to Figures 1E,F, a well-separated TiO_2_ NTs with an inner diameter varying between 97 and to 206 nm and a length of 1.25 μm can be formed in the fluoride-containing EG electrolyte carrying 20 wt% H_2_O. At this percentage, the nanotubular structure is formed in the two phases (α + β) via the formation of an oxide layer (TiO_2_) and the chemical dissolution of this layer assisted by an electric field. The formation mechanism of TiO_2_ NTs from alloys is similar to that of the pristine TiO_2_ NTs obtained from anodization of pure Ti.

#### Composition Analysis

**Figure 2:**
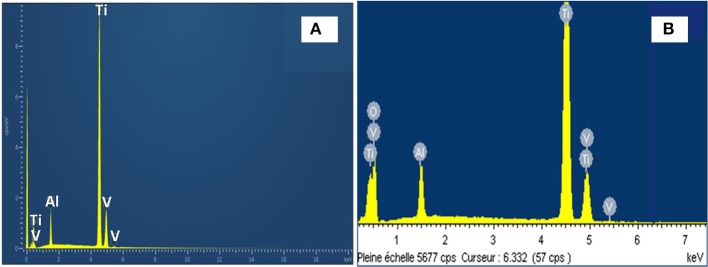
EDS spectra of: pristine TA6V alloy **(A)** and anodized TA6V alloy in fluoride ethylene glycol electrolyte with 20 wt% H_2_O content **(B)**.

Figure 2 shows the EDS spectra of TiO_2_ NTs grown from anodization of TA6V alloy with 20 wt% H_2_O content in the fluoride EG. The energy dispersive X-ray characterization values are summarized in Table 1. After anodization, there is a slight difference of wt% for each element and the strong presence of oxygen confirming the formation of oxides. In fact, the wt% of Ti in the anodized TA6V alloy is lower compared to that of the pristine TA6V alloy. This result suggests that Ti anode is oxidized into TiO_2_ through the oxidation of Ti to form Ti^4+^ according to Equation (1).

(1) $$\begin{array}{l} \text{Ti}\to \text{T}{\text{i}}^{4+}+4{\text{e}}^{-} \end{array}$$

(2) $$\begin{array}{l} {\text{H}}_{2}\text{O}\to 2{\text{H}}^{+}+{\text{O}}^{2-} \end{array}$$

(3) $$\begin{array}{l} \text{T}{\text{i}}^{4+}+2{\text{O}}^{2-}\to \text{Ti}{\text{O}}_{2} \end{array}$$

(4) $$\begin{array}{l} \text{T}{\text{i}}^{4+}+2{\text{H}}_{2}\text{O}\to 4{\text{H}}^{+}+\text{Ti}{\text{O}}_{2} \end{array}$$

**Table 1:** Elemental composition of the pristine TA6V alloy and the anodized TA6V alloy obtained in fluoride ethylene glycol electrolyte carrying 20 wt% H_2_O content.

**Element**	**% Weight**		**% Atomic**	
	**Pristine TA6V alloy**	**Anodized TA6V alloy**	**Pristine TA6V alloy**	**Anodized TA6V alloy**
Al	6.41	3.61	10.87	3.95
V	3.21	2.68	2.88	1.55
O	–	29.93	–	55.21
Ti	90.37	63.78	86.25	39.29

The dissociation of H_2_O takes place at the cathode (Equation 2) and the overall reaction leads to the formation of the titanim dioxide (Equations 3 and 4) (Ortiz et al., 2009). TiO_2_ NTs formed in the fluoride electrolyte are characterized by different morphological parameters (diameter, thickness, length,…) that can be confirmed from Figures 1E,F.

The EDS analysis also shows that Al and V values are lower compared to the TA6V alloy, which can be explained by the oxidation of these elements to form the thin oxide layers of Al_2_O_3_ and V_2_O_5_ (or VO_2_), respectively (Gibran et al., 2018). Note that Al and V signals may come also from the bulk alloy, not only from the surface oxide layer (Benea et al., 2014). The presence of these layers improves the osseointegration and enhances the biocompatibility of the implant material (Jo et al., 2013). It can be noted that no trace of F can be detected suggesting that this element is not incorporated into the oxides during the anodization process.

### Structural Properties

**Figure 3:**
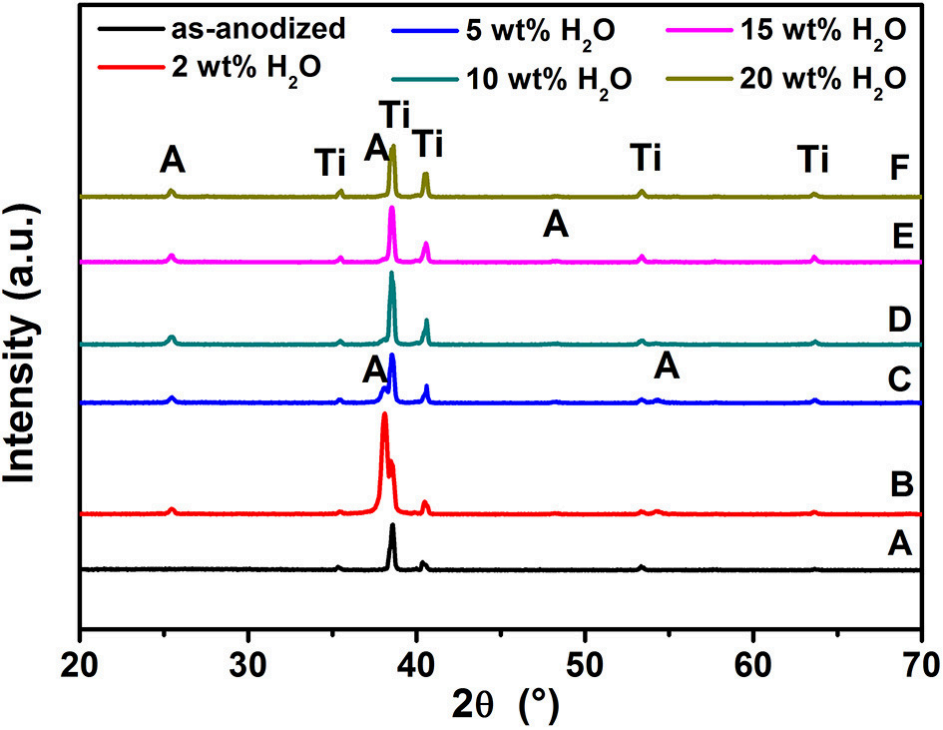
XRD patterns of TiO_2_ NTs grown on TA6V alloy: as-anodized **(A)**, films annealed at 500°C for 3 h with the water content in the electrolyte is: 2 wt% H_2_O **(B)**, 10 wt% H_2_O **(C)**, 5 wt% H_2_O **(D)**, 15 wt% H_2_O **(E)**, and 20 wt% H_2_O **(F)**.“A” is Anatase, “Ti” is substrate of film.

After anodization, the as-formed TiO_2_ NTs at various water contents were annealed at 500°C for 3 h to convert the amorphous compound into a crystalline structure. Figure 3 shows the XRD patterns of these films. Compared to the as-anodized TiO_2_ NTs using 20 wt% H_2_O, the crystallizations of the TiO_2_ NTs films are mainly composed of anatase phase, as evidenced by the diffraction peaks at 2θ = 25.50, 37.80, 48.30, and 55.10°. The diffraction peaks can be indexed to the (101), (004), (200), and (211) planes, respectively (JCPDS Pattern no 00-021-1272). Furthermore, the XRD patterns give no indication of the presence of the Al_2_O_3_ and V_2_O_5_ (or VO_2_) peaks due to their low percentage in the samples and the high dispersion of metal ions in the nanotubular lattice (Li et al., 2009; Tang et al., 2014).

### Optical Properties

**Figure 4:**
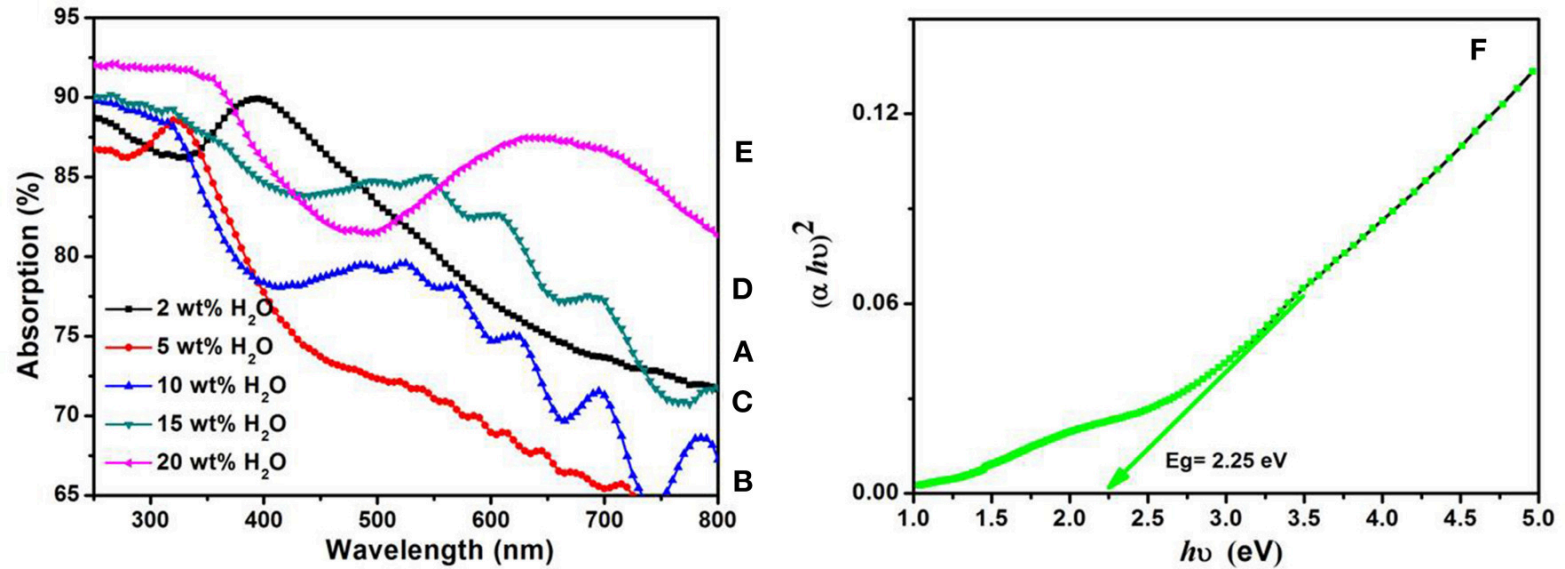
UV-Vis absorption spectra of annealed TiO_2_ NTs layers grown from anodization of TA6V alloys at 60 V for 3 h in fluoride ethylene glycol electrolyte with different water contents: 2wt%H_2_O **(A)**, 5wt%H_2_O **(B)**, 10wt%H_2_O **(C)**, 15wt%H_2_O **(D)**, and 20wt%H_2_O **(E)**. Variations of *(*α*h*υ*)*^2^ vs. photon energy *(h*υ*)* of TiO_2_ NTs synthesized with 20 wt% H_2_O in the fluoride ethylene glycol electrolyte **(F)**.

Figures 4A–E shows the optical absorption spectra of the annealed TiO_2_ NTs at 500°C for 3 h obtained from anodization of TA6V alloy in fluoride EG electrolyte at different water contents. The strong absorptions of these films in the range of 250–336 nm correspond to the electron-transition from the valence band (VB) to the conduction band (CB) with creation of two very reactive species, an electron in the CB and a hole in the VB (Hoffmann et al., 1995). The UV absorption edge of samples prepared using 2, 5, 10, 15, and 20 wt% H_2_O that are around 332, 335, 340, 380, and 410 nm, respectively, correspond to the maximum absorption edge for each curves that are projected on the wavelength axis (nm). By increasing the water content in the solution, the absorption band extends into the visible region. This behavior explained by the increase of the active surface (number of reaction sites) with the formation of TiO_2_ NTs characterized by an improvement of their morphological parameters (diameter, thickness, length…) confirmed by the SEM images given in Figures 1E,F. In addition, the presence of vanadium oxide in the structure is responsible for additional impurity states in the band gap near the CB or the VB altering the optical properties of the material (Li et al., 2009; Nešić et al., 2013; Chen et al., 2015). The same behavior was obtained by the study of Luo et al. suggesting that the extends of absorption edge into the visible region is attributed to the quantum size effects (Luo et al., 2008).

Evaluation of the TiO_2_ NTs band gap (*Eg*) grown from anodization of Ti- 6Al- 4V alloy can be obtained from the absorption coefficient α given in Equation (5) (Mane et al., 2005)

(5) $$\begin{array}{l} \text{α}={\left(\text{hυ}-\text{Eg}\right)}^{0,5}/\text{hυ} \end{array}$$

**Figure 5:**
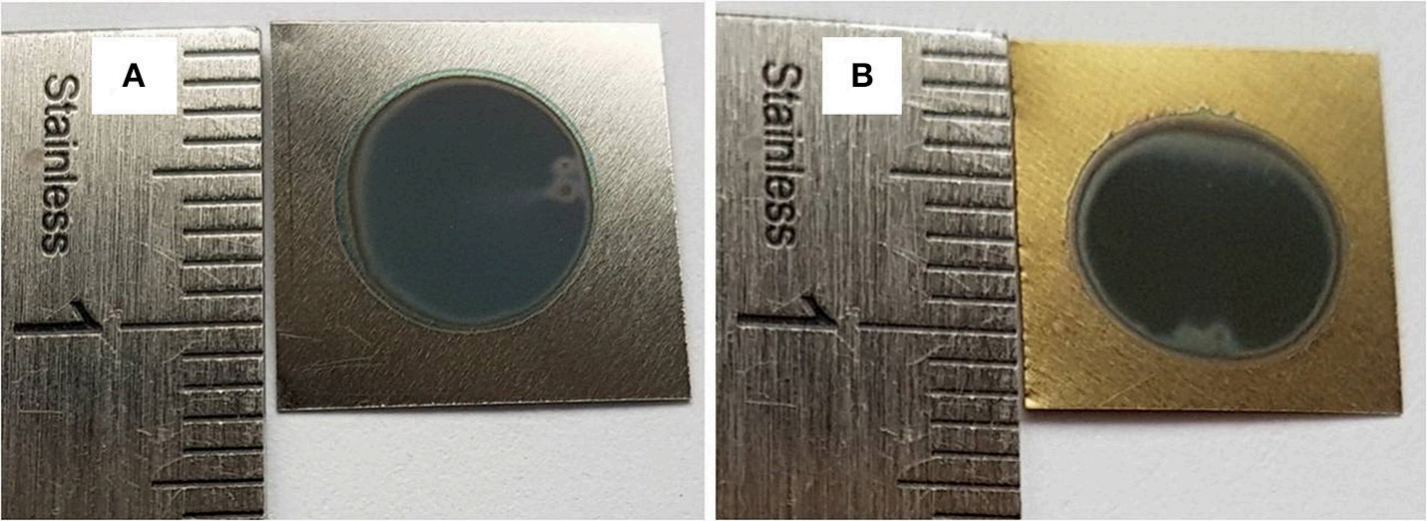
Ti-6Al-4V alloys anodized in fluoride ethylene glycol electrolyte with 20 wt%H_2_O at 60V for 3 h: as-formed **(A)** and annealed at 500 °C for 3 h **(B)**.

α = A / 1, where A is the absorption film, l is the tubes length (l = 1.25 μm) and *h*υ is the photon energy. Figure 4F shows the variations of *(*α*h*υ*)*^2^ vs. photon energy (*h*υ) for the film synthesized using 20 wt% H_2_O in the electrolyte. The extrapolation of the straight line to zero absorption gives the effective band gap energy (*Eg*) value of approximately 2.25 eV, which is significantly lower than that of TiO_2_ anatase (~3.2 eV) (Li et al., 2013). The low band gap value is explained by the presence of vanadium oxide that can extend the absorption band into the visible region. Compared to the color of as-formed Ti−6Al−4V alloy in Figure 5A, this result is in agreement with the EDS analysis and the appearance of yellow-green color in the annealed sample at 500°C for 3 h as shown in Figure 5B. The color reflected on the annealed TiO_2_ NTs sample at 20 wt% H_2_O can be determined through the UV absorption spectra. As seen, the lowest absorption spectra is approximately 500 nm, reflecting a yellow-green color. In the agreement with the previous findings, the V-doped TiO_2_ materials were prepared by both sol-gel technique and liquid phase deposition (LPD) reported that the presence of V can widen the absorption threshold wavelength to 650 nm (Gu et al., 2007; Zhou et al., 2010). In Figure 5A is presented a photo of the sample before annealing.

### Electrochemical Performance

**Figure 6:**
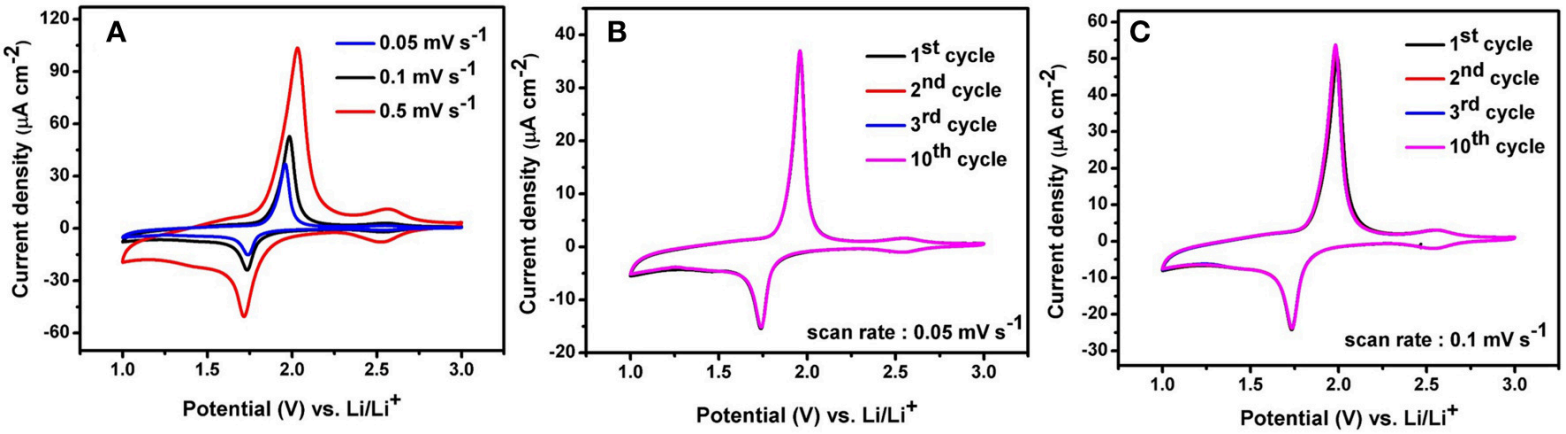
Cyclic voltammograms of anatase TiO_2_ NTs on Ti−6Al−4V alloy performed in the potential range 1–3 V at a scan rate of 0.05 mV.s^−1^,0.1 mV.s^−1^, and 0.5 mV.s^−1^ **(A)**, 10 cycles CVs at scan rate of 0.05 mV.s^−1^**(B)** and 10 cycles CVs at scan rate of 0.1 mV.s^−1^**(C)**.

In this work, self-organized TiO_2_ NTs fabricated from TA6V alloy containing α and β phase are investigated as a potential anode materials for Li-ion microbatteries. The electrochemical performance of TiO_2_ NTs synthesized using ethylene glycol electrolyte containing 20 wt% H_2_O is elucidated through cyclic voltammetry (CV) to analyse the charging and discharging mechanisms during cycling. The anodic and cathodic peaks obtained during the measurements represent the possible phase transformations or redox reactions with the electrodes (Heinze, 1984). The TiO_2_ NTs on TA6V alloy was tested at various scanning rates (0.05, 0.1 and 0.5 mV.s^−1^) between 1 and 3 V vs. Li/Li^+^ at room temperature, as displayed in Figure 6A. Two distinct cathodic and anodic peaks are observed for all scan rates, corresponding to the lithium insertion (Ti^4+^ → Ti^3+^) and extraction (Ti^3+^ → Ti^4+^) in anatase (Li et al., 2012). At a scan rate of 0.05 mV.s^−1^, the cathodic peak centered at ~1.74 V vs. Li/Li^+^ and the anodic peak centered at 1.96 V vs. Li/Li^+^ show a peak potential separation (ΔEp) of 0.22 V. The cathodic and anodic peak slightly shifted to 1.73 V vs. Li/Li^+^ and 1.97 V vs. Li/Li^+^, respectively at 0.1 mV.s^−1^, showing the ΔEp of 0.24 V. Further higher scan rate of 0.5 mV.s^−1^, the cathodic and anodic peaks are significantly shifted to ~1.71 V vs. Li/Li^+^ and ~2.03 V vs. Li/Li^+^, respectively with the ΔEp of 0.32 V. Obviously, as the scan rate was increased, the displacement current increased due to the fact that the over potential become higher.

The separation between the cathodic and anodic peaks indicated the extent of polarization. Hence, the slow scan rate is selected to establish an electrochemical equilibrium between the active species due to the fast scan rate might provoke peak identification more difficult (Plylahan et al., 2015). In addition to the main peaks, an additional peak pair at a potential of ~2.55 V vs. Li/Li^+^ with a low current density is showed up in the reduction and oxidation potential at three different scan rates. It is due to the presence of an electrochemically active vanadium oxide with low valence state of vanadium such as VO_2_ phase (Mai et al., 2010; Mattelaer et al., 2017). The formation of nanotubes on two phases (α + β) titanium alloys leads to the selective dissolution of the elements and the different reactions rates at different phases, yielding VO_2_ and Al_2_O_3_ phases. Considering the peak intensity of VO_2_ with respect to anatase TiO_2_ NTs, it is assumed that the VO_2_ phase might not contribute significantly to the storage performance of the electrode. Apart from both peak pairs, no additional peak for the Al_2_O_3_ phase can be detected in the CVs curves as this phase is probably to be electrochemically inactive. Furthermore, cyclic voltammograms at low scan rate of both 0.05 and 0.1 mV.s^−1^ for 10 cycles reveal a good stability of the electrode which is attested by no peak shifting (see Figures 6B,C). The main cathodic and anodic peaks can be clearly identified up to the 10th cycle.

**Figure 7:**
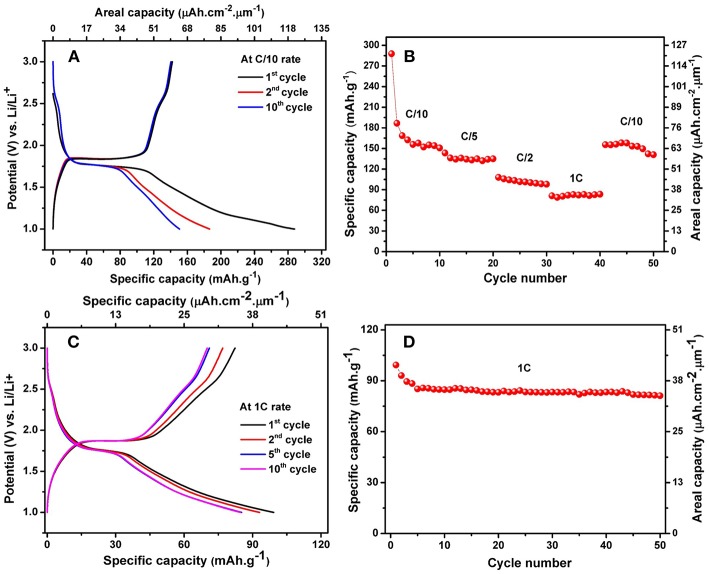
Charge-discharge profile of anatase TiO_2_ NTs on Ti−6Al−4V alloy at C/10 rate **(A)** and the discharge capacity vs. cycle number at multiple C-rates **(B)**, Charge-discharge profile of anatase TiO_2_ NTs on Ti−6Al−4V alloy at 1C rate **(C)** and cycling performance of anatase TiO_2_ NTs on Ti−6Al−4V alloy at 1C rate **(D)**.

The charge-discharge behaviors of the alloyed TiO_2_ NTs were examined by galvanostatic tests between cut-off voltages of 1 and 3 V vs. Li/Li^+^. The results are shown in Figure 7A. It is found that in the first discharge process, there is a short plateau at ~2.55 V which indicates a very small amount of Li ions inserted into VO_2_ phase with a low storage capacity. This plateau is in good accordance with the cyclic voltammogram curve. The potential continuously drops and reaches the large constant plateau at ~1.77 V which is attributed to homogeneous Li insertion into the bulk anatase with a lithium insertion capacity of about 85 mAh.g^−1^ (36 μAh.cm^−2^.μm^−1^). The slope after the plateau, started from ~1.77 V to 1 V has the insertion capacity of 183 mAh.g^−1^ (77 μAh.cm^−2^.μm^−1^), which is attributed to the energy capacity accumulated on the surface of anatase. The lithium extraction capacity is solely about 20 mAh.g^−1^ within the charging potential window of 1–1.84 V in the first cycle, which is smaller than the capacity in the discharging potential region of 1.77–1 V. This results indicates that the irreversible capacity mainly occurs within the sloped region between 1.77 and 1 V. However, the main voltage plateaus consist of the discharge plateau at ~1.77 V vs. Li/Li^+^ and a charge plateau at ~1.84 V vs. Li/Li^+^, resulting in a very small polarization of 0.07 V at C/10 rate. In a good agreement with previous results, the charge plateau at ~1.89 V vs. Li/Li^+^ and discharge plateau at ~1.75 Vvs. Li/Li^+^ with a higher polarization of 0.14 V at C/10 rate are obtained for the self-organized TiO_2_ NTs synthesized in a solution of ethylene glycol containing 1.0 wt% H_2_O and 2 wt% NH_4_F (Prosini et al., 2013). The smaller difference of the charge and discharge plateaus indicates the better electrode reaction kinetics and better rate performance. The reversible Li^+^ insertion into TiO_2_ NTs can be written according to Equation (6) (Djenizian et al., 2011).

(6) $$\begin{array}{l} \text{Ti}{\text{O}}_{2}+\text{xL}{\text{i}}^{+}+\text{x}{\text{e}}^{-}↔\text{L}{\text{i}}_{\text{x}}\text{Ti}{\text{O}}_{2} \end{array}$$

Taking the middle points between these two plateaus, the average working potentials of cell were determined to be ~1.80 V vs. Li/Li^+^. The high working potential of the nanotubes is an advantage to avoid the electrolyte reduction and limit the formation of a solid electrolyte interphase (SEI) layer on the surface of the electrode (Xu et al., 2007).

The electrochemical reactionat the anode is based on the reduction of Ti^4+^ to Ti^3+^ and the Li^+^ insertion into the TiO_2_ NTs. Lithium ions can be inserted reversibly into anatase TiO_2_ to form Li_0.5_TiO_2_, giving a theoretical specific capacity of 168 mAh.g^−1^while the theoretical capacity of amorphous TiO_2_ NTs is 335 mAh.g^−1^ for the insertion of one Li per Ti unit (Auer et al., 2018). In this study, the obtained initial discharge and charge capacities of the electrodes are 288 mAh.g^−1^ (122 μAh.cm^−2^.μm^−1^) and 142 mAh.g^−1^ (60 μAh.cm^−2^.μm^−1^), corresponding to the lithium insertion coefficient of 0.86 and 0.42, respectively with a relatively low initial coulombic efficiency of 49.30%. A high capacity of anatase TiO_2_ NTs in the first few initial cycles is probably due to the presence of the remaining amorphous nanotubes (Prosini et al., 2013).

For the subsequent cycles, the discharge capacity values recorded in the 2nd and 10th cycles are 186 and 150 mAh.g^−1^ with an improved coulombic efficiency of 76.34 and 94.67%, respectively. The capacity fading from the 2nd to the 10th cycle can be attributed to an irreversible reaction of Li^+^ ions with OH groups existing on the surface of nanotubes at low voltages (Ferrari et al., 2017). In addition, the initial capacity loss may also be caused by the interfacial reaction between the residual traces of water on the surface of the nanotubes and lithium salt in the electrolyte combined with the presence of structural defects (Hanzu et al., 2011; Chung et al., 2015). However, the cycling retention continuously improved after first few cycles, thereby coulombic efficiency approaches 100%.

The cells were cycled at multiple C-rates as presented in Figure 7B. TiO_2_ NTs electrode gives a stable capacity of 150 mAh.g^−1^ (63 μAh.cm^−2^.μm^−1^) at C/10, 134 mAh.g^−1^ (56μAh.cm^−2^.μm^−1^) at C/5, 101 mAh.g^−1^ (43 μAh.cm^−2^.μm^−1^) at C/2 and 83 mAh.g^−1^ (35 μAh.cm^−2^.μm^−1^) at 1C. The capacity can be recovered after cycling at C/10 rate over 50 cycles.We noted that the capacity loss at C/10 rate after cycling at fast kinetic rates is attributed to the hindered migration of Li^+^ ions within the TiO_2_ NTs system due to the presence of other crystalline phases. However, it can be observed that the capacity of two last cycles are enough stable at C/10 rate, hence we assumed the discharge capacities stabilize after few initial cycles. To prove the cycling stability of the TiO_2_ NTs, galvanostatic charge-discharge were performed at 1C rate up to 50 cycles (Figures 7C,D). The results thus clearly show a good cycling stability of the TiO_2_ NTs electrodes that can deliver a reversible capacity of 82 mAh.g^−1^ (34 μAh.cm^−2^.μm^−1^).

## Conclusion

In summary, self-organized TiO_2_ NTs have been successfully synthesized via anodization of Ti−6Al−4V alloy at 60 V for 3 h in fluoride ethylene glycol electrolyte at various water contents (2 wt% up to 20 wt% H_2_O). Significant differences in morphological structure of TiO_2_ NTs were obtained. At low water content, a honeycomb and porous structure is formed on the surface due to the presence of both α and β phases in the Ti−6Al−4V alloy leading to a dissimilar non-uniform etching. Remarkably, self-organized TiO_2_ NTs could be formed uniformly across both α and β phases at 20 wt% H_2_O. The optical properties and electrochemical performance of the anodized TiO_2_ NTs carrying 20 wt% H_2_O have been investigated. The anatase TiO_2_ NTs offers a low band gap value equal to 2.25 eV due to the presence of vanadium oxide in the structure that widens the threshold of absorption wavelength into the visible region. Moreover, galvanostatic charge-discharge tests exhibited a good capacity of 82 mAh.g^−1^ (34 μAh.cm^−2^.μm^−1^) at 1C rate over 50 cycles. These results show that the self organized TiO_2_ NTs grown from TA6V alloy can be considered as competitive anode materials for Li-ion microbatteries, as well as other potential applications in gas sensors, solar cells, and photocatalysis.

## Author Contributions

HF and VS performed experiments, analyzed the experimental results, and wrote the manuscript. DH, MB, FV, ME, MP, and TD discussed experimental results. All the authors contributed to the reading of paper and gave advice on the revision of the manuscript.

### Conflict of Interest Statement

The authors declare that the research was conducted in the absence of any commercial or financial relationships that could be construed as a potential conflict of interest.
